# W-Net: Convolutional neural network for segmenting remote sensing images by dual path semantics

**DOI:** 10.1371/journal.pone.0288311

**Published:** 2023-07-27

**Authors:** Guangjie Liu, Qi Wang, Jinlong Zhu, Haotong Hong

**Affiliations:** 1 College of Computer Science and Technology, Changchun Normal University, Changchun, Jilin, China; 2 FAW Mold Manufacturing Co., Ltd, Changchun, Jilin, China; University of Wisconsin-Eau Claire, UNITED STATES

## Abstract

In the latest research progress, deep neural networks have been revolutionized by frameworks to extract image features more accurately. In this study, we focus on an attention model that can be useful in deep neural networks and propose a simple but strong feature extraction deep network architecture, W-Net. The architecture of our W-Net network has two mutually independent path structures, and it is designed with the following advantages. (1) There are two independent effective paths in our proposed network structure, and the two paths capture more contextual information from different scales in different ways. (2) The two paths acquire different feature images, and in the upsampling approach, we use bilinear interpolation thus reducing the feature map distortion phenomenon and integrating the different images processed. (3) The feature image processing is at a bottleneck, and a hierarchical attention module is constructed at the bottleneck by reclassifying after the channel attention module and the spatial attention module, resulting in more efficient and accurate processing of feature images. During the experiment, we also tested iSAID, a massively high spatial resolution remote sensing image dataset, with further experimental data comparison to demonstrate the generality of our method for remote sensor image segmentation.

## Introduction

Traditional deep learning-based networks solve the problem of learning features directly from data by using a small surrounding area a pixel as the CNN input to perform training and prediction, which is inefficient and inaccurate [1, 2]. A fully convolutional neural network (FCN) is the first pixel-level semantic segmentation method that outputs pixel-level classification results directly from arbitrarily sized inputs by upsampling layers within the network, which significantly improves the semantic segmentation results [3].

In the design of neural networks, researchers have focused on leveraging existing network structures, such as VGG, ResNet, ResNeXt, and DenseNet, to extract deep features. The above network structure was originally applied to the image classification problem, and the extracted features represent semantics [4–7]. Networks such as U-Net, GoogLeNet, SegNet, PSPNet, and DeepLab have been proposed to achieve more prominent results in semantic segmentation tasks through model improvements or module innovations [8–12].

As compared to other natural images, remote sensing images have a larger image scale and more small target objects, and the background and foreground imbalance background information is more complex and variable. Moreover, the traditional semantic segmentation algorithm has poor applicability, low segmentation accuracy, and blurred target boundaries. Whether we can design a new semantic segmentation for remote sensing images and obtain better segmentation results is a very challenging problem. We investigate a novel network architecture, called W-Net, which solves the above problem. We find that FCN can take input images of any size, that Image spatial information is preserved in the end-to-end implementation of segmented feature images, and that we can recover the class to which each pixel subsumes from these abstract features; U-Net uses a feature stitching approach, which contains more features in each dimension and can reuse shallow features with high spatial resolution. We propose a dual-path convolution module based on FCN and U-Net. After running through two independent paths, the images are sampled at different sizes and the feature images of multi-scale images are fused to have both a global field of perception and detailed information small about target objects. The attention mechanism is introduced into the model bottleneck. Instead of processing all images, the spatial attention model and channel attention model are combined to select fused feature maps in a targeted manner. Attention is focused on specific parts of the image, key information in the image is extracted while irrelevant information is ignored, the number of network layers is not too deep, and the performance of the whole model is improved.

### Contribution

Our main contributions are three-fold.

(1) A new dual path network structure, W-Net, is proposed for remote sensing image segmentation.

(2) The design and effectiveness of the proposed dual-path convolution module and focus on prominent areas module in W-Net are verified by adequate ablation studies.

(3) Experiments are conducted on the largest remote sensing dataset iSAID to verify the generalization of W-Net.

## Related works

Following the continuous innovation of deep learning technology, deep learning methods have been widely used in image processing fields such as object detection and image segmentation [13, 14]. In much research, it has been shown that an advanced neural network architecture is one of the most challenging and effective ways to enhance image segmentation task performance. However, compared to traditional methods, from the first truly deep learning semantic segmentation model, the FCN extends end-to-end convolutional networks to semantic segmentation, and the high-performance network structure shines in semantic segmentation tasks.

### General semantic segmentation

The most intuitive way to enhance the performance of a network is to deepen the network structure by overlaying more layers, thereby allowing the network to extract image features more accurately. The design of VGG and ResNet follows exactly this approach. Compared to AlexNet, VGG has more than twice the depth [15]. In addition, ResNet has 22 times more layers than VGG. ResNet presents the first residual network structure using a constant mapping layer *y* = *x* with the output equal to the input. The problems of gradient disappearance caused by increasing the depth of the network structure are alleviated. DenseNet also uses a connection of different feature mappings. It guarantees the transmission of information, makes the network narrower and less parameterized, and connects all the layers together. GoogLeNet is also very deep but presents an inception module [16–18]. It stops increasing the model depth and starts feature extraction from the model by changing the model width. It uses filters of different sizes on each convolutional block to highlight different features in the same layer, allowing the network to show higher performance.

As the depth of the network structure continues to increase, new improvements to the network structure must be proposed to further address the accuracy of the feature maps. In the FCN, upsampling is performed using a deconvolutional layer, and the roughness of upsampling is improved by jump connections. Its fully connected layer structure is still used in the most advanced segmentation model. Rough segmentation maps are produced by the upper convolutional layers and some jump connections, and the FCN introduces more jump connections to improve the effect. However, the FCN network only replicates the encoder features, whereas the proposed SegNet network transfers the maximum pooling index to the decoder, thereby improving the resolution of the segmented images. This results in SegNet being more memory-efficient than FCN. To increase the appropriate perceptual field index without reducing spatial dimensionality, DeepLab v1 uses dilated convolution. It proposes a pyramidal collection of voids in spatial dimensions and substitutes the fully-connected layer in VGG with a convolutional layer to extract a combined picture of multidimensional features using a fully-connected conditional random field (CRF). Global scene classification is also important because it provides clues for segmenting the category distribution, and the pyramid pooling module proposed by the PSPNet network uses a large kernel pooling layer to capture this information. Images with different inputs (of different sizes) are filtered using convolution kernels of different sizes, and then the output is upsampled to restore the identical in size to the input, making the contextual information of the features more obvious. The DPN network is the first dual-path model that studies the advantages and limitations of ResNeXt and DenseNet, proposing a dual-path architecture to enrich path design. The structure of DPN is not too complex as it merges two networks through an inception-style structure, which is essentially a model integration approach, and is even more effective for image classification [19]. U^2^-Net is a fancy new network structure proposed on the basis of U-Net, which is modeled on the coding–decoding structure. A new module, ReSidual U-blocks (RSU), has been tested and has achieved remarkable results for segmenting the foreground of objects [20].

### Remote sensing image segmentation

Semantic segmentation is widely used in remote sensing images, primarily using general semantic segmentation methods applied to specific areas such as road detection, crop yield estimation, and land change monitoring, and many improved techniques are also available [21–24]. Recently, FarSeg proposed a relational and optimization-based foreground modeling approach to solve the false alarm and foreground-background imbalance problems [25]. However, the performance of small object segmentation suffers from the lack of edge information used to distinguish adjacent object features. PFNet is based on the PFN framework to insert PFM between feature pyramids, and the constructed pyramidal propagation network solves the problems of uneven distribution of front and rear views of aerial images and numerous small objects under high resolution [26].

### Research objectives

For object classification and localization, the traditional model uses only the feature maps from the last layer of the feature extraction network, resulting in a large downsampling rate. However, this is not applicable to remote sensing images with complex foreground information and a wide variety of small target objects. We propose a multi-scale feature fusion through a dual path composed of U-Net and FCN, and a mechanism for focusing attention on salient regions. In contrast, our method produces effective suppression of irrelevant background regions, solves the problems of poor foreground perception of small target objects in remote sensing images and ineffective propagation of semantic features at the edges of objects, and provides better model performance and results.

## Proposed method

**Fig 1:**
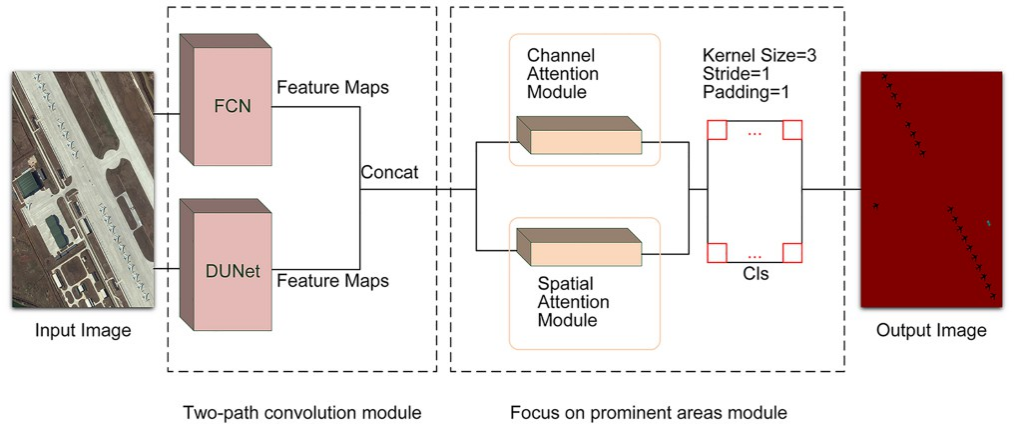
The W-Net network structure consists of two modules: Two-path convolution module and focus on prominent areas module.

To extract high-resolution image features more effectively and make the model more focused on foreground features, we designed a W-Net network structure with the structure diagram shown in Fig 1. There are two modules that make up our semantic segmentation network: the first is a convolutional module composed of dual paths for extracting image features, and the second is a focused salient feature region module used to improve the accuracy of feature images after image classification. In Section 3.1, we introduce the two-path convolution module. In Section 3.2, the design of the network structure is introduced.

### Two-path convolution module

#### Fully convolutional network (FCN)

The FCN replaces the fully connected layer of the traditional CNN with a convolutional layer and proposes a jump connection to the convolutional layer to enhance the roughness of upsampling. An FCN without fully connected layers can accommodate arbitrarily sized inputs, but the output of such a network is a heat map rather than a category. At the same time, to solve the smaller image size due to convolution and pooling, the image size is recovered using upsampling, but the obtained results are not sufficiently fine and not sensitive enough to detail.

#### U-Net

This is a variant of an FCN, which increases the sensory field, it focuses on global features and more on local features, such as texture. While extracting features with each downsampling, some edge features are inevitably lost. By upsampling, of course, a larger feature maps can be obtained, but the edges of the feature map are missing information and upsampling does not retrieve these lost features.

#### Two-path convolution module

**Fig 2:**
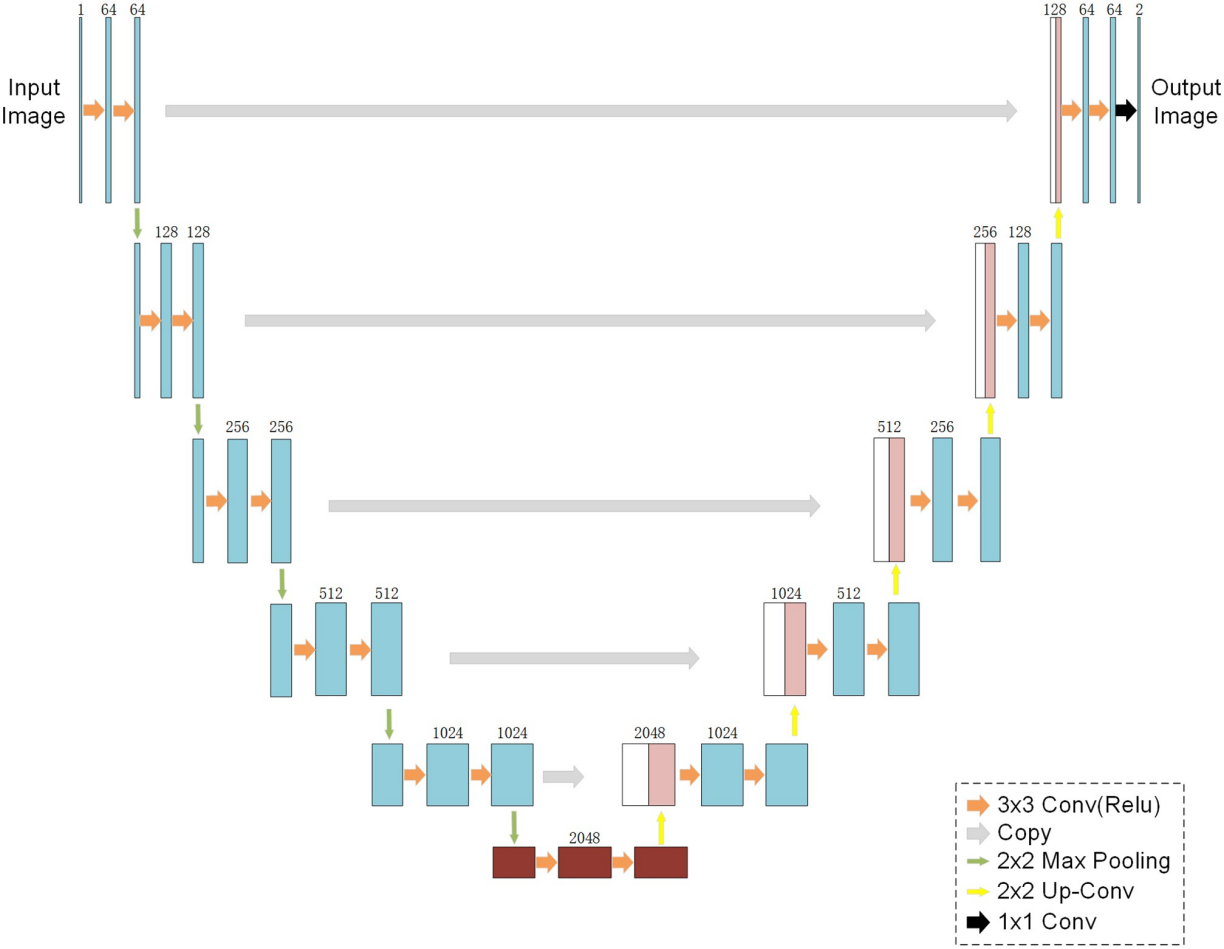
U-Net has poor ability to handle remote sensing images with excessive contextual information. DUNet obtains better segmentation results by deepening the network structure.

We propose a model of a two-path convolutional block, as shown in Fig 1. We refer to the FCN solution for global and local information. It is defined as a jump structure that exploits a feature spectrum that incorporates deep, coarse semantic information and shallow, fine-grained representational information to produce accurate and fine-grained segmentation. It outputs feature information as a heat map, and although the result is not sufficiently fine, it effectively preserves the edge information of the feature map. By comparison with the FCN, U-Net performs a subtle multi-scale feature integration and ensures that the feature acquisition positions are not shifted. U-Net performs exceptionally well on medical images and obtains pixel-level fine segmentation. However, in the process of continuous upsampling, the edge information of complex images cannot be effectively classified. Compared to medical images, remote sensing images contain a larger number of objects and more complex foreground information. Therefore, we study the advantages and disadvantages of the two models, consider fine-tuning the U-Net network structure, and propose a deeper U-Net network structure, DUNet, as shown in Fig 2. The feature maps obtained by connecting the two networks after feature extraction through two independent paths are then subjected to feature classification, thus solving more effectively the problem of inaccurate extraction of edge information from high-resolution image feature maps.

**Fig 3:**
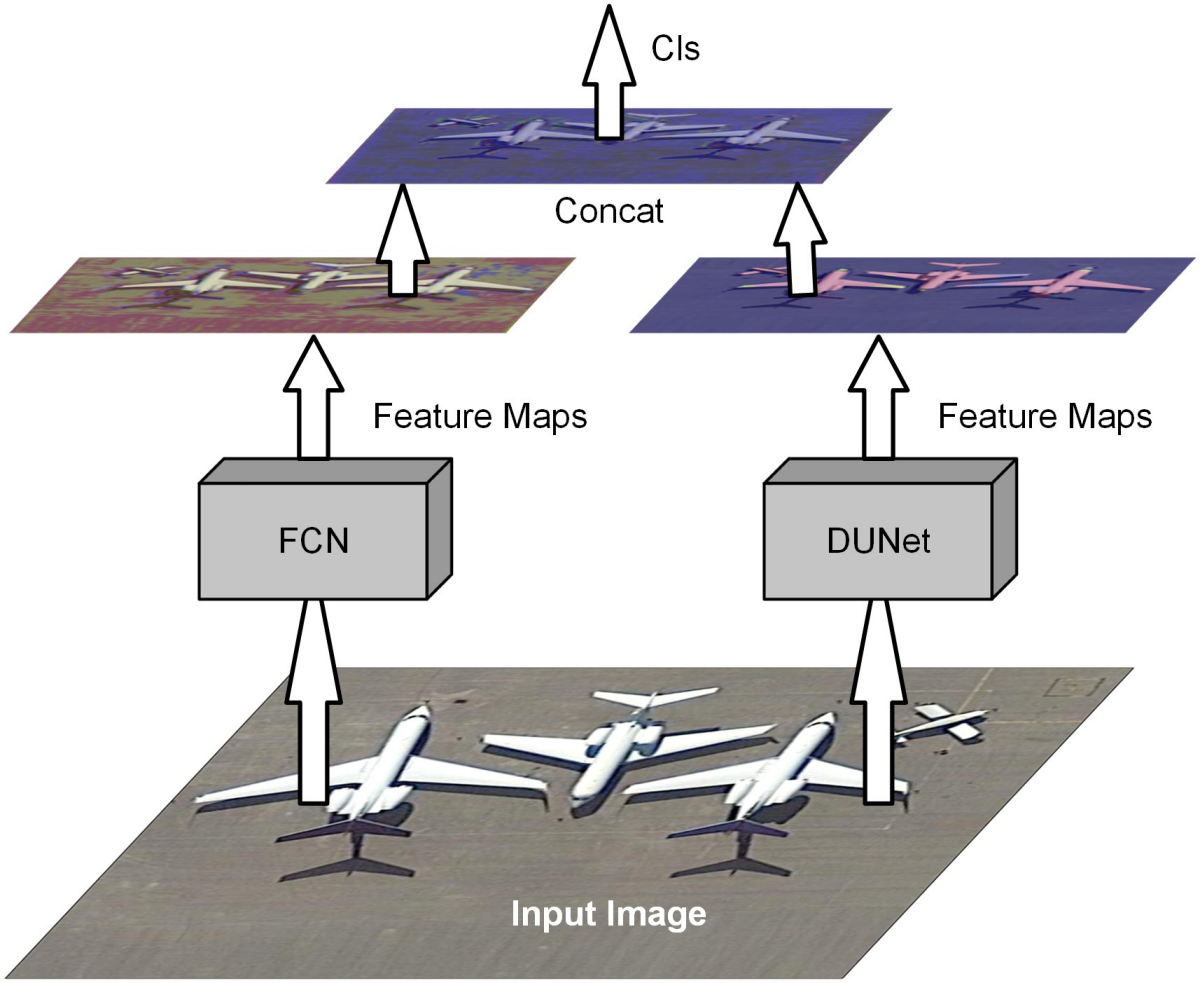
The two-path convolution module contains two independent paths of FCN and DUNet for feature extraction of the image, and the fused image retains more feature information.

The structure of the two-path convolution module is shown in Fig 3. In the two-path convolution module, the image is independently feature extracted through two paths. The feature map obtained by connecting the two feature maps after feature extraction is then subjected to feature classification, thus solving the problem of inaccurate extraction of edge information in the feature map of high-resolution images more effectively.

### Focus on prominent areas module

HSR remote sensing images have many object categories and differences in the size of different object scales, as well as problems of misalignment and overlap. Therefore, it is particularly important to focus attention on the region of significant features. In the dual-path convolution module, we extract feature maps containing more effective boundary information, at which point we focus on the impact of attentional mechanisms on deep neural networks in general. The central purpose of an attention mechanism is to obtain a network that focuses on what needs more attention. It has a variety of implementations, but the core of each of them is attention [27]. Attentional mechanisms can be divided into channel attentional mechanisms, spatial attentional mechanisms, and a combination of both. Our study suggests that the model can be enhanced by adding an attentional mechanism combining the channel attentional mechanism and spatial attentional mechanism at the bottleneck of the network structure. Therefore, we propose the focus on prominent areas module that contains the attention mechanism and image classification operation. An attention-focused information range is obtained by this module, and it effectively solves the problem of focusing on the foreground features of remote sensing images.

## Experiments

### Experimental setting

#### Datasets

*Training dataset.* Training data were collected by the Jilin Institute of High-Resolution Remote Sensing, and these images were obtained from the Landsat-7 platform. The original image size ranged from ∼ 800 * 800 pixels to ∼ 4000 * 13000 pixels for a total of 31 HSR remote sensing images. After the data enhancement process, a predefined training and validation set was divided into 8665 images containing three classes using the dataset.

*Evaluation datasets.* The iSAID dataset consisted of 2806 HSR remotely sensed images [28]. These images were acquired at multiple resolutions and from multiple sensors and platforms. The size of the original images ranged from ∼ 800 * 800 pixels to ∼ 4000 * 13000 pixels. The iSAID dataset contains 15 classes with a total of 655451 target instances, and the number of instances in a single image can reach up to 8000, with an average of 239. There are 1411 images in the pre-training set, 458 images in the validation set, and 937 images in the test set of iSAID. This is the largest dataset of HSR remotely sensed image segmentation in the direction of remote sensing image segmentation so far.

#### Evaluation metrics

*Mean intersection over union (mIoU).* According to the commonly available assessment methods, we utilize the average cross-merge rate as the major evaluation index of object segmentation in the following way:

$$\begin{array}{c} mIoU=\frac{1}{K}\sum_{i=1}^{K}\frac{P⋂G}{P⋃G}, \end{array}$$ (1)

where *P* is the predicted value, *G* is the true value, and *K* is the total number of categories. Overall, the formula represents the intersection of the predicted and true values of each category divided by the concurrent set and then averaged [29–31].

*Pixel accuracy (PA).* Pixel-based accuracy calculation is the most basic and simplest of the evaluation metrics. *PA* is the number of pixels correctly predicted as a percentage of the number of total pixels and is evaluated as follows:

$$\begin{array}{c} PA=\frac{\sum_{i=0}^{K}p_{ii}}{\sum_{i=0}^{K}\sum_{j=0}^{K}p_{ij}}, \end{array}$$ (2)

where *i* denotes the true value, *j* denotes the predicted value, *K* denotes the total number of categories, and p_ij_ denotes the number of pixels that predict *i* to *j*.

#### Implementation details

The data were enhanced during the training process. We scaled and cropped each image to 512 * 512. The backbone network used for the FCN path in the dual-path convolution module of W-Net was HRNet_W18, which was pre-trained on ImageNet. DUNet is not using any existing backbone network and must be trained from scratch. We used the SGD optimizer to optimize our network, with the weight decay set to 0.0005 and the momentum set to 0.9. In all the experiments, these models were trained using a “poly” learning rate strategy $\left(base\_lr*{\left(1-\frac{epoch}{max\_epoch}\right)}^{power}\right)$ for 40k iterations, where *base*_*lr* = 0.01 and *power* = 0.9. To assure that the output feature map was the same size as the input image, we used bilinear interpolation for all upsampling processes. Our network was implemented based on PaddlePaddle2.0.2, using a server with a 4-core CPU and a Tesla V100 GPU for training, with a batch image count of 8 during 40k iterations of training [32].

### Comparison with general methods

**Fig 4:**
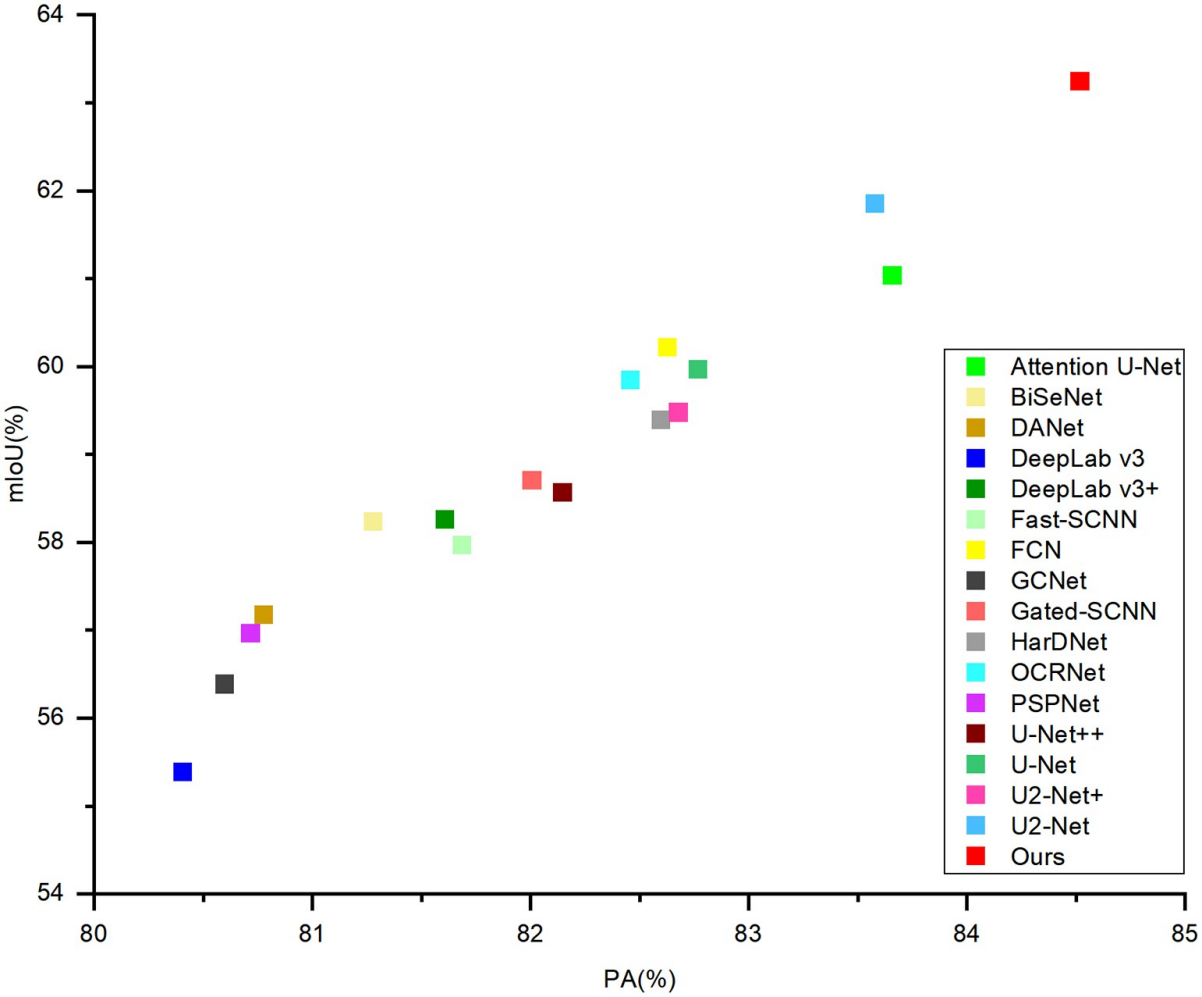
Accuracy (PA) vs. precision (mIoU) on the training set.

In order to assess the effectiveness of W-Net more comprehensively, in Table 1 we compare W-Net with several CNN-based approaches for comprehensive experimental results. From classical models to state-of-the-art models, including Attention U-Net, BiSeNet, DANet, DeepLab v3, DeepLab v3+, Fast-SCNN, FCN, GCNet, Gated-SCNN, HarDNet, OCRNet, PSPNet, U^2^-Net, U^2^-Net+, U-Net, and U-Net++ [33–44]. The quantitative results in the comparison presented in Table 1 indicate that W-Net outperformed the other methods in high-resolution scenarios. We compare with 16 classical and advanced image segmentation methods at the present stage. According to the mIoU evaluation index, the result of our model is increased by 1.39% to 7.86% compared with other methods. The experimental results on the segmented dataset show that the method is more effective than the existing general semantic segmentation methods, and the segmentation results obtained are more accurate. Fig 4 demonstrates the accuracy and precision. This reveals that W-Net achieves an effective improvement in accuracy and precision, which benefits from efficient and precise module design.

**Table 1:** The accuracy of each model in object segmentation and the mIoU results are shown on the training set by qualitative experiments. The best results in the experiments are indicated by the values in bold in each column.

Method	PA(%)	mIoU(%)	IoU per category(%)			
			Background	Rice	Corn	Forest
Attention U-Net	83.66	61.03	74.83	35.20	48.77	85.31
BiSeNet	81.28	58.23	71.26	35.98	42.03	83.65
DANet	80.78	57.17	71.09	33.67	41.14	82.79
DeepLab v3	80.41	55.38	70.94	28.76	39.97	81.83
DeepLab v3+	81.61	58.25	72.77	34.88	42.60	82.74
Fast-SCNN	81.69	57.96	71.60	32.59	43.80	83.85
FCN	82.63	60.21	73.08	38.78	43.40	85.58
GCNet	80.6	56.38	70.91	32.53	41.03	81.06
Gated-SCNN	82.01	58.70	73.89	30.99	48.00	81.89
HarDNet	82.60	59.39	72.66	33.94	46.06	84.89
OCRNet	82.46	59.84	72.83	37.19	46.30	83.05
PSPNet	80.72	56.96	71.05	31.04	40.47	82.19
U-Net++	82.15	58.56	73.29	32.28	44.33	84.36
U-Net	82.77	59.96	74.31	33.62	48.90	83.00
U^2^-Net+	82.68	59.47	73.56	33.21	45.23	85.87
U^2^-Net	83.58	61.85	74.32	**39.05**	48.41	85.63
Ours	**84.52**	**63.24**	**75.71**	38.94	**52.38**	**85.92**

### Ablation studys

For verifying the validity of W-Net, sufficient experiments were conducted to investigate the ablation of network modules in relation to important parameters.

#### Two-path convolution module

*FCN path.* We need to consider the backbone network that the FCN path uses. To verify the accuracy of the backbone network segmentation on remote sensing images, we replaced different backbones to perform ablation studies. The HRNET backbone is more richly expressed semantically and more precisely spatially [45]. It can maintain high-resolution feature representation throughout the process, allowing high-resolution remote sensing images to retain feature information better. Therefore, as presented in Table 2, we conducted experiments using HRNet. The results show that the HRNet_W18 version reached 60.21% mIoU during training. Although the mIoU of HRNet_W32 reaches 59.69%, there was a noticeable increase in the size and calculation of the model as the number of channels increased. Therefore, we confirmed that an increase in the number of channels provides no advantage for the determination of the FCN backbone. We also tested the lightweight models HRNet_W18_Small_V1 and HRNet_W18_Small_V2, and found reduced accuracy. Therefore, we determined that using HRNet_W18 is the most effective FCN backbone for deep high-resolution representation learning.

**Table 2:** Accuracy (PA), precision (mIoU), and consistency (Kappa) obtained by testing using different backbones in the fully convolutional neural network (FCN).

Method	backbone	mIoU(%)	PA(%)	Kappa
FCN	HRNet_W18	**60.21**	**82.63**	**0.7296**
	HRNet_W32	59.69	82.22	0.7250
	HRNet_W44	58.40	81.97	0.7199
	HRNet_W48	50.48	75.21	0.6255
	HRNet_W60	58.90	81.97	0.7196
	HRNet_W64	59.55	81.95	0.7206
	HRNet_W18_Small_V1	56.73	80.58	0.7007
	HRNet_W18_Small_V2	58.82	82.16	0.7232

*DUNet path.* As listed in Table 3, the mIoU value of the U^2^-Net network is 61.85%. U^2^-Net is a new module called ReSidual U-blocks (RSU) proposed on top of U-Net, which can segment the object foreground well and exceed the segmentation results of the U-Net network. The mIoU of the U-Net network is 59.96%, and it is obvious from Fig 4 that U-Net attains the top ranking in terms of accuracy and precision. The DUNet network we propose to solve the more complex situation of information elements in remote sensing images is significantly better than U^2^-Net in the modified DUNet structure that we tested to obtain the results. This confirms that our model effectively solves the aforementioned problems and that the U-Net-based model structure is effectively enhanced.

**Table 3:** Data comparison of the proposed DUNet network modules in terms of mIoU, PA, and Kappa.

Method	mIoU(%)	PA(%)	Kappa
U-Net++	58.56	82.15	0.7228
U-Net	59.96	82.77	0.7325
U^2^-Net+	59.47	82.68	0.7312
U^2^-Net	61.85	83.58	0.7455
DUNet	**62.40**	**84.07**	**0.7528**

*Two-path convolution module.* Tables 2 and 3 clearly indicate that the FCN model with the HRNet_W18 backbone and the newly proposed DUNet model achieves the highest value of mIoU, using the same parameters. To further verify the correctness of the concept of the dual-path convolution module, we formed a dual path by combining the two methods. An image was obtained as two feature images by the input of two paths, and then the feature maps were integrated. To understand the content of images more precisely, we must apply image classification (cls) such that meaningful tasks can be extracted from the images. The dual path uses an FCN with DUNet and applies cls. The mIoU results in Table 4 verify that cls is very useful after feature map integration. Therefore, we believe that our newly designed dual-path convolution module outperforms FCNs and U-Net in this image-segmentation task.

**Table 4:** In contrast to the use of FCN, U-Net, and DUNet methods for determining dual paths, verifying that image classification (cls) must be applied for the image segmentation task. The values in bold font indicate the best type of dual path convolution module.

Method	mIoU(%)	PA(%)
FCN	60.21	82.63
U-Net	59.96	82.77
FCN+U-Net	58.34	81.97
FCN+U-Net+cls	62.39	84.04
FCN+DUNet	59.92	82.46
FCN+DUNet+cls	**63.13**	**84.29**

#### Focus on prominent areas module

We conducted experiments by ablation to verify the role of BAM’s proposed dilation value (*d*) and reduction ratio (*r*) in focusing on the significant region module. The results are presented in Table 5. In the BAM, in the ablation experiment, the setting of the expansion value d did not affect the parameter size, but the error was minimal for *d* = 4 [46]. Therefore, we fixed *d* = 4 and studied the effectiveness of the reduction ratio r on the two-path convolution module. With an mIoU of 63.24%, *r* = 4 is the optimal solution for the focused significant region module. In summary, the hyperparameter *r* of BAM of focus on prominent areas module in W-Net is set to 4.

**Table 5:** Experimental results of the optimal solution for the reduction ratio.

*r*	2	4	8
mIoU(%)	62.50	**63.24**	63.09

#### Experimental parameter optimization

**Fig 5:**
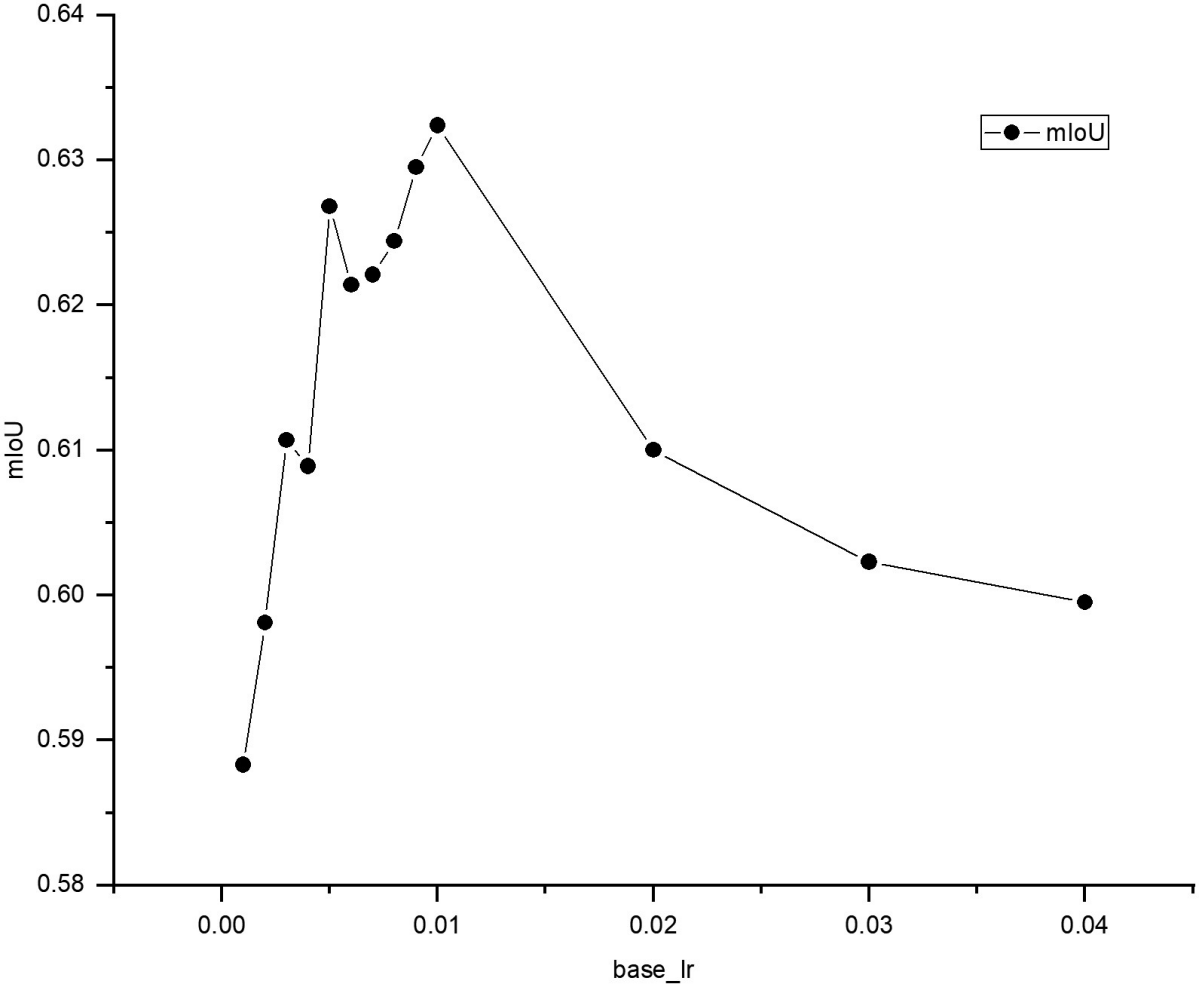
mIoU (%) for W-Net optimization using different*base*_*lr*.

It is important to confirm that the learning rate set in the gradient descent process can effectively control the update step in each iteration of the algorithm. Further, the problem that a learning rate set too large is prone to oscillation and set too small is too slow to converge should be solved. Therefore, we tuned the important hyperparameter *base*_*lr* in the poly-learning strategy and used different *base*_*lr* values for our experiments. We have better displayed the experimental results in Fig 5 by means of a line graph. As *base*_*lr* increases, the performance continuously improves. Using *base*_*lr* = 0.01, the optimization of W-Net improves the mIoU by 1.1% over the baseline, bringing the mIoU to 63.24%. However, when *base*_*lr* = 0.02, the performance decreases. Therefore, we set the hyperparameter *base*_*lr* in the learning rate to 0.01 in the model.

### Qualitative comparison

To understand the performance of our model more intuitively, we conducted comprehensive experiments on a larger-scale HSR remote sensing image dataset, iSAID. We contrasted W-Net with U-Net and U-Net’s related methods U-Net++, U^2^-Net, with species from classical to state-of-the-art methods, including Fast-SCNN, DNL-Net, FCN, FastFCN, DeepLabv3, DeepLabv3+, PSPNet, DDRNet, GINet, HRNet, and FarSeg [47–51]. In Table 6, the quantitative results can clarify that the segmentation results of W-Net in HSR remote sensing images are significantly better than other methods with an mIoU of 63.68%. The IoU obtained by each method is compared in Table 7 for each category. In the table, W-Net shows excellent results in 11 of the 15 categories.

**Table 6:** Semantic segmentation of the HSR remote sensing dataset iSAID dataset using state-of-the-art methods to compare the experimental mIoU.

Method	backbone	mIoU(%)
Fast-SCNN	-	47.63
U-Net++	-	48.58
U-Net	-	49.25
DNL-Net	ResNet50	49.44
FCN	HRNet_W18	50.96
FastFCN	ResNet50	55.10
U^2^-Net	-	56.61
DeepLab v3	ResNet50	59.61
DeepLab v3+	ResNet50	60.21
PSPNet	ResNet50	60.29
DDRNet	-	61.35
GINet	ResNet50	61.64
HRNet	HRNet_W18	62.10
FarSeg	ResNet50	63.54
W-Net	HRNet_W18	**63.68**

**Table 7:** The bold values in each column indicate the IoU best results for each type of object segmentation on the iSAID dataset. Each abbreviation is explained as: SV(Small vehicle), BD(Baseball diamond), HC(Helicopter), SP(Swimming pool), TC(Tennis court), LV(Large vehicle), SC(Storage tank), GTF(Ground field track), SBF(Soccer-ball field), BC(Basketball court), and RA(Roundabout).

Method	mIoU(%)	IoU per category (%)														
		SV	BD	TC	Ship	LV	SC	SP	Bridge	GTF	HC	Plane	BC	Harbor	SBF	RA
Fast-SCNN	47.63	45.78	43.16	53.08	76.73	26.81	25.11	8.28	51.88	26.36	1.37	37.42	17.68	45.75	66.66	34.90
U-Net++	48.58	43.12	37.31	52.99	69.24	27.35	31.16	5.75	49.81	24.76	0	40.95	29.67	47.37	66.75	43.66
U-Net	49.25	48.66	46.84	43.86	81.71	35.85	20.99	10.07	53.78	25.92	0	38.34	0	54.41	71.63	44.39
DNL-Net	49.44	51.11	51.55	41.31	81.95	32.07	19.98	20.60	51.37	39.78	0	38.63	0.22	38.93	76.24	49.28
FCN	50.96	50.86	50.51	56.13	80.12	30.42	30.80	13.77	53.96	31.33	0.49	37.27	24.00	53.99	69.78	43.99
FastFCN	55.10	53.91	53.02	48.07	85.70	41.07	27.89	22.99	57.29	41.26	0	40.79	22.96	43.24	76.86	49.31
U^2^-Net	56.61	52.17	50.91	63.83	79.15	35.34	37.62	16.44	52.24	32.47	1.99	44.63	28.20	59.37	67.75	39.69
Deeplabv3	59.61	55.05	51.02	59.87	77.92	33.43	34.88	17.63	53.84	33.59	2.05	47.23	24.35	60.56	70.61	48.17
Deeplabv3+	60.21	52.67	55.67	64.10	83.02	38.08	39.60	24.35	52.60	31.82	4.55	34.69	33.04	59.07	70.94	46.45
PSPNet	60.29	60.82	56.60	68.94	86.93	44.64	42.51	27.46	58.25	40.13	6.38	44.74	41.05	64.44	77.56	52.55
DDRNet	61.35	61.95	58.13	66.19	88.09	47.80	42.78	26.28	59.31	39.69	14.73	46.79	32.77	65.19	78.09	54.41
GINet	61.64	60.31	58.04	67.28	86.97	50.37	39.37	**32.35**	59.00	41.84	3.28	49.67	**52.64**	62.97	78.16	51.91
HRNet	62.10	58.82	**62.45**	69.56	86.49	46.83	43.15	30.73	57.23	39.52	13.39	44.85	45.88	64.79	76.70	51.15
FarSeg	63.54	63.71	58.77	70.43	87.84	49.62	**44.98**	23.23	60.66	41.13	4.23	43.78	38.57	67.51	78.72	52.15
W-Net	**63.68**	**64.77**	59.74	**72.11**	**88.91**	**55.9**	42.09	29.50	**61.54**	**43.18**	**18.62**	**50.62**	44.41	**67.75**	**79.97**	**56.68**

**Fig 6:**
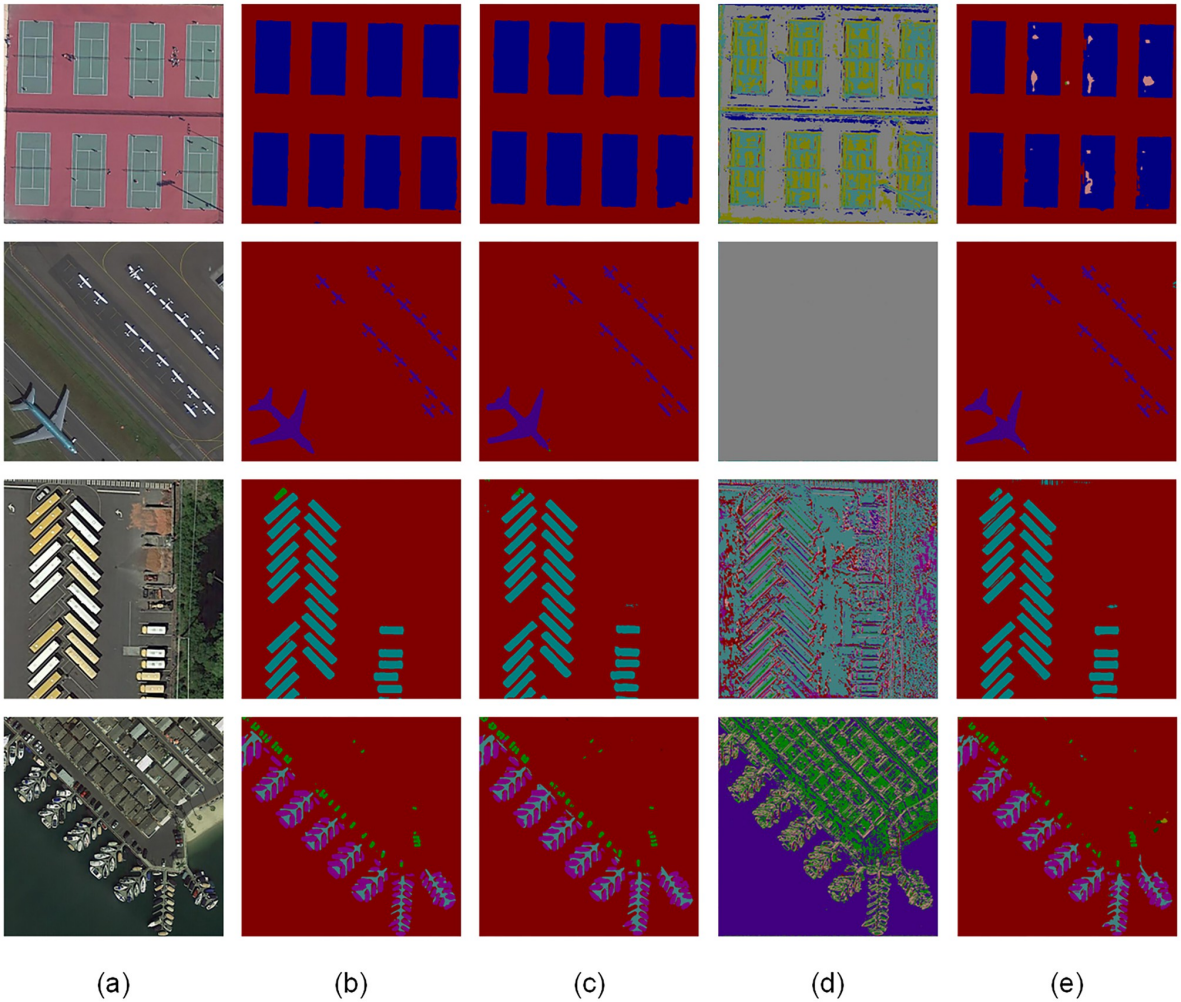
Qualitative experiments based on the use of the W-Net network on the iSAID test set were compared with three other methods. (a) iSAID test set image, (b) W-Net network object segmentation map, (c) U^2^-Net network object segmentation map, (d) FCN network object segmentation map, (e) U-Net network object segmentation map. To facilitate visualization of the mapping relationships of the objects of the experimental results, we resize the images. Legend: Scene 1 (tennis court), Scene 2 (airplane), Scene 3 (small vehicle, large vehicle), Scene 4 (small vehicle, port, ship).

Fig 6 shows a plot of our results for U^2^-Net, FCN, and U-Net. In the first row of the results plot, you can see the effect of segmentation when there is a shadow occlusion problem of other objects. As we can observe, our W-Net produces accurate results in the presence of shaded occluders. The U^2^-Net model appears to have less accurate objects owing to shadows, and FCN and U-Net have larger errors. Rows 2 and 3 show the images of both large and small objects. Our model can simply and effectively distinguish between different forms of the same class of objects, although the edges of small objects are blurred. U^2^-Net and U-Net clearly showed inaccurate segmentation edges for small objects and two markers for one object. The FCN failed to segment objects. Row 4 shows the power of our model to segment objects with cluttered backgrounds and complex foregrounds. In conclusion, our model can effectively solve the challenges in image segmentation by performing multi-scale contextual feature extraction on HSR remote sensing images to produce highly accurate image segmentation results.

## Conclusion

In this paper, we consider the problem of edge information extraction processing of feature maps and complex backgrounds as a solution challenge for HSR remote sensing image segmentation, which is ignored by general semantic segmentation methods. To overcome these questions, we propose a W-Net network structure. It extracts the feature map from multiple scales by means of a dual-path convolution structure and further refines the boundary information of the feature map by adding a focused significant region module. The comprehensive experimental results indicated that W-Net is efficient in HSR remote sensing image segmentation and exceeds the general methods in terms of accuracy mIoU.

Although our model solves the above problems, the size of the model affects the speed. To find a better way to strike a balance between speed and accuracy, we will continue to investigate different advanced techniques and model architectures in the future.
